# Wer nutzt Videosprechstunden in einer psychiatrischen Institutsambulanz? Demografische Analyse des Einsatzes von Videosprechstunden aus Abrechnungsdaten zwischen 2021 und 2023

**DOI:** 10.1007/s00115-025-01809-7

**Published:** 2025-02-13

**Authors:** Matthias Nieberler, Felix Das, Anna Linda Leutritz, Bodo Warrings

**Affiliations:** 1 Klinik und Poliklinik für Psychiatrie, Psychosomatik und Psychotherapie, Universitätsklinikum Würzburg, Margarete-Höppel-Platz 1, 97080 Würzburg, Deutschland; 2 Neurologische Klinik und Poliklinik, Universitätsklinikum Würzburg, Würzburg, Deutschland

## Hintergrund

Seit 2017 sind ärztliche und psychotherapeutische Konsultationen im Rahmen von Videosprechstunden als Regelleistung zulasten der gesetzlichen Krankenversicherung abrechenbar, erst zu Beginn der COVID-19-Pandemie gelangten sie jedoch zunehmend in den Fokus der öffentlichen Wahrnehmung. 2020 wurden Videosprechstunden auch in die Vergütungsvereinbarung bayerischer psychiatrischer Institutsambulanzen (PIAs) nach § 118 SGB V aufgenommen, welche ein multiprofessionelles Behandlungsangebot für schwer akut und chronisch psychisch Erkrankte darstellen.

Dass die Behandlung von Patienten^1^ mithilfe von Videosprechstunden effizient und sicher ist, konnte mittlerweile durch Untersuchungen aus verschiedenen medizinischen Bereichen gezeigt werden. In der Behandlung von Menschen mit Depression, Angststörungen und posttraumatischer Belastungsstörung erreichen Videosprechstunden im Vergleich mit der konventionellen Behandlung vor Ort ähnliche Ergebnisse in der therapeutischen Wirksamkeit, Patientenzufriedenheit und Therapieabbruchquote [5].

Demografische Analysen zu Patienten, die das Angebot von Videosprechstunden in Anspruch nehmen, sind in Deutschland spärlich. 2021 betrug der Anteil weiblicher Patienten unter den Nutzern von Videosprechstunden über alle Fachbereiche hinweg 63 %. 61 % der Nutzer waren maximal 40 Jahre alt, 78 % lebten im städtischen Raum [2].

Ziel dieser Untersuchung war es, die Nutzergruppe von Videosprechstunden in unserer PIA nach demografischen und klinischen Gesichtspunkten näher zu charakterisieren und mit der Gruppe, die das Angebot von Videosprechstunden nicht wahrnimmt, zu vergleichen.

## Methodik

Routineabrechnungsdaten der PIA am Universitätsklinikum Würzburg wurden retrospektiv ausgewertet. Der Beobachtungszeitraum reichte vom 01.04.2021, dem Datum der Einführung des Angebots von Videosprechstunden, bis zum 31.12.2023.

Insgesamt nahmen 211 Patienten das Angebot mindestens einer Videosprechstunde wahr, welche über die abgerechneten Ziffern PIA130–136 und PIA230–236 identifiziert wurden. Diese Patientengruppe wurde analysiert hinsichtlich Geschlecht, Alter, Entfernung des Wohnortes zur Klinik und Diagnosen. Hierbei wurde bei Patienten, die in verschiedenen Jahren das Angebot von Videosprechstunden wahrnahmen, das Alter zum Zeitpunkt des ersten Videokontaktes gewertet. Alle für die Patienten verschlüsselten psychiatrischen Diagnosen wurden nach ICD-10 in die diagnostischen Kapitel F0x bis F9x eingeteilt.

Dieser Gruppe gegenübergestellt wurden alle konventionellen Vor-Ort-Patientenkontakte eines repräsentativen Quartals (3. Quartal 2021), welche sich auf 479 Patienten verteilten. 39 hiervon, für die sowohl Video- als auch Vor-Ort-Kontakte abgerechnet wurden, wurden aus der Kontrollgruppe entfernt, wodurch 440 Patienten verblieben, welche im 3. Quartal 2021 ausschließlich eine Vor-Ort-Behandlung erfuhren und hinsichtlich der oben genannten Charakteristika äquivalent analysiert wurden.

Die Gruppe von Patienten mit mindestens einer Videosprechstunde wurde mit der Gruppe von Patienten ohne Videosprechstunden verglichen. Die statistische Analyse wurde mit SPSS Statistics (IBM, Armonk, NY, USA) durchgeführt. Unterschiede zwischen den beiden Gruppen wurden mittels Student’s t‑test bzw. Welch’s t‑test (bei ungleichen Varianzen) auf ihre Signifikanz geprüft. Ein *p*-Wert < 0,05 wurde als signifikant angesehen.

## Ergebnisse

Der Anteil von Videosprechstunden an allen abgerechneten PIA-Kontakten lag im Jahr 2021 bei 5,6 %, im Jahr 2022 bei 4,5 % und im Jahr 2023 bei 5,0 % und zeigte somit keine merkliche zeitliche Dynamik. Auch ein Einfluss der COVID-19-Pandemiephasen konnte nicht beobachtet werden. Die demografischen Charakteristika der Nutzer von Videosprechstunden sind denen der Kontrollgruppe in Tab. 1 gegenübergestellt. Signifikante Unterschiede fanden sich für das Geschlecht sowie das Alter. Videosprechstunden-Patienten waren häufiger weiblich und jünger mit einem Unterschied von 11,15 [9,06; 13,23] Jahren. 80,6 % der Videosprechstunden-Patienten waren 40 Jahre oder jünger verglichen mit 43,2 % der Patienten aus der Kontrollgruppe. Der Anteil der Patienten über 50 Jahre war demensprechend mit 7,6 % deutlich kleiner als in der Kontrollgruppe (36,7 %).

**Tab. 1:** Demografische Charakteristika der beiden Gruppen mit Angabe von Mittelwerten und Standardabweichungen sowie *p*-Werten

	Videosprechstunden-Patienten	Kontrollgruppe	*p*-Wert
Geschlecht (weiblich:männlich)	81,0 %:19,0 %	64,8 %:35,2 %	< 0,001
Alter (Jahre)	35,13 ± 10,03	46,27 ± 16,95	< 0,001
Entfernung (km)	37,70 ± 57,90	29,31 ± 54,39	0,079
Anzahl Diagnosen	1,35 ± 0,64	1,27 ± 0,51	0,101

Videosprechstunden-Patienten lebten 8,39 [17,52; 0,75] km weiter entfernt von der Klinik, wobei dieser Unterschied keine Signifikanz zur Kontrollgruppe erreichte. Der Vergleich der Anzahl der Diagnosen verblieb ebenso ohne signifikante Differenz, sodass davon ausgegangen werden kann, dass eine psychiatrische Multimorbidität keinen Einfluss auf die Nutzung von Videosprechstunden hatte.

**Abb. 1:**
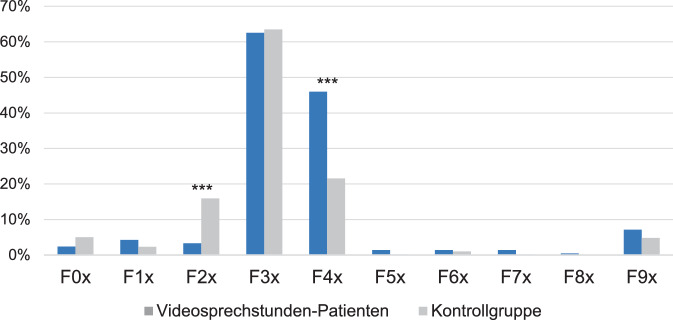
Anteil der Patienten mit mindestens einer Diagnose aus dem entsprechenden ICD-10-Kapitel an allen Patienten der untersuchten Gruppe. *** *p* < 0,001

Vergleicht man die beiden Gruppen weiter, so fällt auf, dass die Patienten, die Videosprechstunden in Anspruch nahmen, signifikant seltener an einer Diagnose aus dem Kapitel F2x der ICD-10 litten, welches psychotische Störungen umfasst (*p* < 0,001). Dagegen war unter den Videosprechstunden-Patienten der Anteil derer, für die mindestens eine Diagnose aus dem Kapitel F4x codiert wurde, signifikant größer (*p* < 0,001). Kapitel F4x umfasst neurotische, Belastungs- und somatoforme Störungen. Für die anderen Kapitel der psychiatrischen Diagnosen im ICD-10 ergaben sich keine signifikanten Unterschiede zwischen den beiden Gruppen. Die Verteilung der Diagnosen ist in Abb. 1 aufgezeigt.

## Diskussion

Neben der digitalen Kompetenz und individuellen Präferenzen der jeweiligen Behandler üben auch patientenbezogene Charakteristika einen starken Einfluss auf den Einsatz von Videosprechstunden aus: Die vorliegende Analyse zeigt, dass weibliche und jüngere Patienten in unserer PIA signifikant häufiger unter Einsatz von Videosprechstunden behandelt werden. Es ist bekannt, dass Frauen und jüngere Menschen mit höherer Wahrscheinlichkeit eine psychiatrisch-psychotherapeutische Behandlung in Anspruch nehmen [4]. Dieser Effekt scheint für die Nutzer von Videosprechstunden in unserer PIA noch ausgeprägter zu sein, was die bisher wenigen deutschen Analysen hierzu zumindest teilweise bestätigen können [2].

Für die Interpretation der Diagnosen muss berücksichtigt werden, dass deren Verteilung stark durch die inhaltlichen Schwerpunkte unserer Klinik bestimmt wird. In unserer PIA werden neben allgemeinpsychiatrischen Patienten schwerpunktmäßig Menschen mit affektiven Störungen, Angststörungen und peripartalen psychischen Störungen behandelt. So ist es auch nicht verwunderlich, dass entsprechende Erkrankungen am häufigsten diagnostiziert wurden. Während der Anteil der Diagnosen aus dem Bereich der affektiven Störungen (F3x) bei den Videosprechstunden-Patienten und der Kontrollgruppe nahezu identisch ist, sind in der Videosprechstunden-Gruppe signifikant häufiger Patienten mit einer Diagnose aus dem Kapitel F4x vertreten (*p* < 0,001). Dieses umfasst im Wesentlichen Angst- und Zwangsstörungen, posttraumatische Belastungsstörungen sowie dissoziative und Somatisierungsstörungen, also klassische psychotherapeutisch zu behandelnde Erkrankungen. Im Gegensatz dazu wurden Patienten mit psychotischen Störungen (F2x) mit signifikantem Unterschied seltener unter Einsatz von Videosprechstunden behandelt (*p* < 0,001). Dies lässt sich mitunter dadurch erklären, dass Videosprechstunden vorwiegend für psychologische Kontakte eingesetzt wurden (75,6 % vs. 24,4 % ärztliche Kontakte) und Patienten mit psychotischen Störungen seltener eine Psychotherapie erhalten [6].

Zudem muss limitierend angemerkt werden, dass bisher keine standardisierte Vorgehensweise zum Einsatz von Videosprechstunden in unserer PIA etabliert war. Bei Erstvorstellung wurde bei Zustimmung des Patienten eine Datenschutzeinwilligung für potenziell stattfindende Videosprechstunden eingeholt. Ob diese dann eingesetzt wurden, unterlag jedoch ausschließlich der individuellen Maßgabe des jeweiligen Behandlers. Es muss also davon ausgegangen werden, dass neben klinischen Kriterien u. a. sowohl die digitale Affinität des Behandlers als auch die vom Behandler vermutete digitale Kompetenz des Patienten einen großen Einfluss auf die Entscheidung für oder gegen Videosprechstunden ausgeübt hat. Der Zugang zur Videosprechstunde erfolgt browserbasiert über einen im Vorfeld per E‑Mail verschickten Link, sodass keine besonderen technischen Anforderungen vorliegen und auch eine mobile Nutzung möglich ist.

Zusammenfassend zeigt die hier vorliegende Untersuchung, dass männliche und/oder ältere Patienten sowie Menschen mit psychotischen Störungen in unserer PIA seltener eine telemedizinische Behandlung mit Videosprechstunden erhalten, wobei sich nicht differenzieren lässt, ob diese Patienten seltener eine virtuelle Behandlung einfordern oder ihnen diese nur weniger häufig angeboten wird. Dabei handelt es sich hier um eine sehr vulnerable Gruppe, die insgesamt seltener eine psychiatrisch-psychotherapeutische Behandlung in Anspruch nimmt und eine sehr hohe Suizidrate besitzt [3].

Um auch diesen Patienten eine telemedizinische Behandlung zu ermöglichen, sollten nicht nur die Behandler sensibilisiert werden, sondern auch standardisierte Vorgehensweisen zum Einsatz von Videosprechstunden entwickelt werden. Es bedarf außerdem mehr gezielter Programme durch Krankenkassen und öffentliche Träger zur breiten Aufklärung über den Zugang zu telemedizinischer Behandlung. Werden Videosprechstunden zukünftig gezielter eingesetzt, so bieten sie das Potenzial vorhandene Versorgungslücken zu verkleinern [1] und so zur Entstigmatisierung psychisch erkrankter Menschen beizutragen.

## Fazit für die Praxis

- Videosprechstunden wurden in unserer Untersuchung vorwiegend von jüngeren und/oder weiblichen Patienten genutzt. Nutzer von Videosprechstunden hatten außerdem häufiger mindestens eine Diagnose aus dem Kapitel F4x und seltener eine Diagnose aus dem Kapitel F2x der ICD-10.
- Auch männlichen, älteren Patienten sowie Menschen mit psychotischen Störungen sollten Videosprechstunden angeboten werden, um diesen den Zugang zu psychiatrisch-psychotherapeutischer Behandlung zu erleichtern.
- Hierfür ist neben öffentlichen Aufklärungsmaßnahmen der Patienten, Angehörigen und Behandler auch die Erarbeitung standardisierter Vorgehensweisen zum Einsatz von Videosprechstunden notwendig.

## Footnotes

1 In dieser Arbeit wird aus Gründen der besseren Lesbarkeit auf die Verwendung der Sprachformen männlich, weiblich und divers verzichtet. Sämtliche Personenbezeichnungen gelten gleichermaßen für alle Geschlechter.
